# [2-(1*H*-Benzimidazol-2-yl-κ*N* ^3^)aniline-κ*N*]dichloridozinc

**DOI:** 10.1107/S1600536811026572

**Published:** 2011-07-09

**Authors:** Naser Eltaher Eltayeb, Siang Guan Teoh, Suchada Chantrapromma, Hoong-Kun Fun

**Affiliations:** a School of Chemical Sciences, Universiti Sains Malaysia, 11800 USM, Penang, Malaysia; b Department of Chemistry, Faculty of Pure and Applied Sciences, International University of Africa, Sudan; c Crystal Materials Research Unit, Department of Chemistry, Faculty of Science, Prince of Songkla University, Hat-Yai, Songkhla 90112, Thailand; d X-ray Crystallography Unit, School of Physics, Universiti Sains Malaysia, 11800 USM, Penang, Malaysia

## Abstract

In the title benzimidazole mononuclear complex, [ZnCl_2_(C_13_H_11_N_3_)], the Zn^II^ ion is four-coordinated in a distorted tetra­hedral geometry by an imidazole N, an amino N and two Cl atoms. The dihedral angle between the benzimidazole and benzene rings is 9.57 (1)°. In the crystal, mol­ecules are linked by weak N—H⋯Cl hydrogen bonds into layers parallel to the *bc* plane. π–π inter­actions with centroid–centroid distances in the range 3.4452 (8)–3.8074 (8) Å are also observed.

## Related literature

For bond-length data, see: Allen *et al.* (1987 ▶). For background to benzimidazoles and their applications, see: Chassaing *et al.* (2008 ▶); Podunavac-Kuzmonovic *et al.* (1999 ▶); Xue *et al.* (2011 ▶). For related structures, see: Eltayeb *et al.* (2007 ▶; 2009 ▶; 2011 ▶); Maldonado-Rogado *et al.* (2007 ▶). For the stability of the temperature controller used in the data collection, see: Cosier & Glazer (1986 ▶).
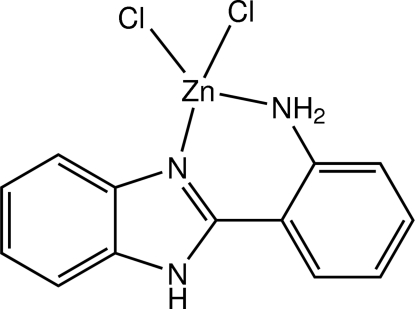


## Experimental

### 

#### Crystal data

[ZnCl_2_(C_13_H_11_N_3_)]
*M* *_r_* = 345.54
Monoclinic, 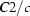

*a* = 22.0252 (7) Å
*b* = 10.0651 (3) Å
*c* = 15.3676 (6) Å
β = 125.244 (2)°
*V* = 2782.32 (18) Å^3^
*Z* = 8
Mo *K*α radiation
μ = 2.14 mm^−1^
*T* = 100 K
0.48 × 0.31 × 0.31 mm

#### Data collection

Bruker APEX DUO CCD area-detector diffractometer
Absorption correction: multi-scan (*SADABS*; Bruker, 2009 ▶) *T*_min_ = 0.428, *T*_max_ = 0.560
50659 measured reflections
7344 independent reflections
5887 reflections with *I* > 2σ(*I*)
*R*_int_ = 0.023

#### Refinement

*R*[*F* ^2^ > 2σ(*F* ^2^)] = 0.027
*wR*(*F* ^2^) = 0.081
*S* = 1.02
7344 reflections
172 parameters
H-atom parameters constrained
Δρ_max_ = 0.54 e Å^−3^
Δρ_min_ = −0.55 e Å^−3^

### 

Data collection: *APEX2* (Bruker, 2009 ▶); cell refinement: *SAINT* (Bruker, 2009 ▶); data reduction: *SAINT*; program(s) used to solve structure: *SHELXTL* (Sheldrick, 2008 ▶); program(s) used to refine structure: *SHELXTL*; molecular graphics: *SHELXTL*; software used to prepare material for publication: *SHELXTL* and *PLATON* (Spek, 2009 ▶).

## Supplementary Material

Crystal structure: contains datablock(s) global, I. DOI: 10.1107/S1600536811026572/rz2622sup1.cif

Structure factors: contains datablock(s) I. DOI: 10.1107/S1600536811026572/rz2622Isup2.hkl

Additional supplementary materials:  crystallographic information; 3D view; checkCIF report

## Figures and Tables

**Table 1:** Hydrogen-bond geometry (Å, °)

*D*—H⋯*A*	*D*—H	H⋯*A*	*D*⋯*A*	*D*—H⋯*A*
N2—H1*N*2⋯Cl1^i^	0.82	2.56	3.3503 (9)	164
N3—H1*N*3⋯Cl1^ii^	0.89	2.49	3.3753 (9)	174
N3—H2*N*3⋯Cl2^iii^	0.94	2.44	3.3015 (12)	153

Symmetry codes: (i) 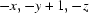; (ii) 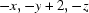; (iii) 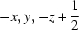.

**Fig. 1.**
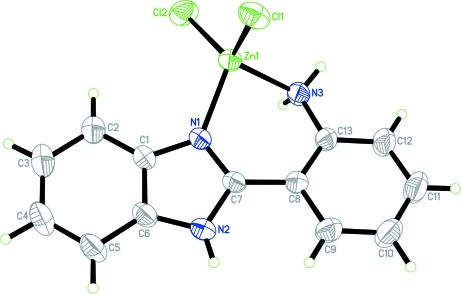
The molecular structure of the title compound, with 50% probability displacement ellipsoids.

**Fig. 2.**
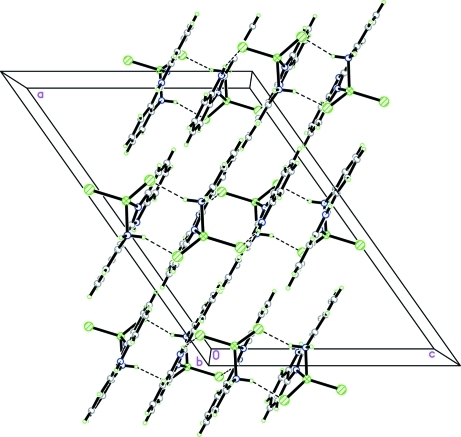
The crystal packing of the title compound viewed down the b axis. Hydrogen bonds are shown as dashed lines.

## supplementary crystallographic information

### Comment

Benzimidazole compounds have a wide range of biological properties such as antibacterial (Chassaing *et al.*, 2008) and inhibitory activity against enteroviruses (Xue *et al.*, 2011). The complexes of transition metal salts with benzimidazole derivatives have been extensively studied as models of some important biological molecules (Podunavac-Kuzmonovic *et al.*, 1999). As part of our ongoing structural studies of benzimidazoles (Eltayeb *et al.*, 2007; 2009; 2011) and as an extension of investigation on their complexes, the title zinc(II) complex, (I), is reported here.

Complex (I) is a mononuclear zinc(II) complex (Fig. 1) in which the coordination geometry around the zinc ion is distorted tetrahedral, with the metal four-coordinated by an imidazole N, an amino N and two Cl atoms. In the complex, the 2-(2-aminophenyl)-1*H*-benzimidazole acts as a bidentate ligand. The bond angles around the central metal zinc(II) show some deviations from ideal tetrahedral geometry [N3-Zn1-Cl1 = 109.27 (3)°, N1-Zn1-Cl2= 114.83 (3)°, Cl1-Zn1-Cl2 = 117.844 (13)° and the bite angle N1–Zn1-N3 = 89.36 (3)°]. The Zn-N [2.0068 (8) and 2.0471 (9) Å] and Zn-Cl [2.2041 (3) and 2.2589 (3) Å] bond lengths are comparable to those found in a similar zinc(II) benzimidazole complex (Maldonado-Rogado *et al.*, 2007). The benzimidazole ring system (C1–C7/N1–N2) is planar (*r.m.s*. of 0.0097 (1) Å), the larger deviation being observed for atom C1 (0.019 (1) Å). The dihedral angle between the benzimidazole and phenyl rings is 9.57 (6)°. The bond lengths of ligand are within normal ranges (Allen *et al.*, 1987).

In the crystal structure (Fig. 2), the molecules are linked through N—H···Cl hydrogen bonds into two dimensional layers parallel to the *bc* plane. π···π interactions are observed with centroid···centroid distances: *Cg*_1_···*Cg*_1_^iii^ = 3.4452 (8)) Å, *Cg*_1_···*Cg*_2_^iii^ = 3.6879 (9) Å and *Cg*_2_···*Cg*_3_^i^ = 3.8074 (8) Å (*Cg*_1_, *Cg*_2_ and *Cg*_3_ are the centroids of the C1/C6–C7/N1–N2, C1–C6 and C8–C13 rings, respectively; symmetry codes: (i) -x, 1-y, -z; (iii) -x, y, 1/2-z).

### Experimental

The title compound was synthesized by adding 2-hydroxy-3-methylbenzaldehyde (0.136 g, 1.0 mmol) to a solution of 2-(2-aminophenyl)-1*H*-benzimidazole (0.209 g, 1.0 mmol) in ethanol (30 mL). The colour of the resulting solution was pale-yellow. Then upon addition of zinc chloride (0.136 g, 1.0 mmol), the colour of the solution became golden-yellow. The mixture was refluxed with stirring for 3 h. The resultant solution was filtered and the filtrate was evaporated to give a yellow solid product. Yellow block-shaped single crystals of the title compound suitable for *X*-ray structure determination were obtained by slow evaporation of an ethanol solution at room temperature after several days.

### Refinement

All H atoms were positioned geometrically and allowed to ride on their parent atoms, with d(N—H) = 0.82 Å for NH; 0.88 and 0.94 Å for NH_2_, d(C-H) = 0.93 Å for aromatic. The *U*_iso_ values was constrained to be 1.2*U*_eq_ of the carrier atoms. The highest residual electron density peak is located at 0.64 Å from Cl1 and the deepest hole is located at 0.49 Å from Cl1. An outliner reflection (2 4 3) was omitted.

### Crystal data

**Table:**

[ZnCl_2_(C_13_H_11_N_3_)]	*F*(000) = 1392
*M**_r_* = 345.54	*D*_x_ = 1.650 Mg m^−^^3^
Monoclinic, *C*2/*c*	Mo *K*α radiation, λ = 0.71073 Å
Hall symbol: -C 2yc	Cell parameters from 7344 reflections
*a* = 22.0252 (7) Å	θ = 2.3–37.7°
*b* = 10.0651 (3) Å	µ = 2.14 mm^−^^1^
*c* = 15.3676 (6) Å	*T* = 100 K
β = 125.244 (2)°	Block, yellow
*V* = 2782.32 (18) Å^3^	0.48 × 0.31 × 0.31 mm
*Z* = 8	

### Data collection

**Table:**

Bruker APEX DUO CCD area-detector diffractometer	7344 independent reflections
Radiation source: sealed tube	5887 reflections with *I* > 2σ(*I*)
graphite	*R*_int_ = 0.023
φ and ω scans	θ_max_ = 37.7°, θ_min_ = 2.3°
Absorption correction: multi-scan (*SADABS*; Bruker, 2009)	*h* = −37→36
*T*_min_ = 0.428, *T*_max_ = 0.560	*k* = −17→17
50659 measured reflections	*l* = −25→26

### Refinement

**Table:**

Refinement on *F*^2^	Primary atom site location: structure-invariant direct methods
Least-squares matrix: full	Secondary atom site location: difference Fourier map
*R*[*F*^2^ > 2σ(*F*^2^)] = 0.027	Hydrogen site location: inferred from neighbouring sites
*wR*(*F*^2^) = 0.081	H-atom parameters constrained
*S* = 1.02	*w* = 1/[σ^2^(*F*_o_^2^) + (0.0419*P*)^2^ + 0.7801*P*] where *P* = (*F*_o_^2^ + 2*F*_c_^2^)/3
7344 reflections	(Δ/σ)_max_ = 0.002
172 parameters	Δρ_max_ = 0.54 e Å^−^^3^
0 restraints	Δρ_min_ = −0.55 e Å^−^^3^

### Special details

**Table:**

Experimental. The crystal was placed in the cold stream of an Oxford Cryosystems Cobra open-flow nitrogen cryostat (Cosier & Glazer, 1986) operating at 100.0 (1) K.
Geometry. All esds (except the esd in the dihedral angle between two l.s. planes) are estimated using the full covariance matrix. The cell esds are taken into account individually in the estimation of esds in distances, angles and torsion angles; correlations between esds in cell parameters are only used when they are defined by crystal symmetry. An approximate (isotropic) treatment of cell esds is used for estimating esds involving l.s. planes.
Refinement. Refinement of F^2^ against ALL reflections. The weighted R-factor wR and goodness of fit S are based on F^2^, conventional R-factors R are based on F, with F set to zero for negative F^2^. The threshold expression of F^2^ > 2sigma(F^2^) is used only for calculating R-factors(gt) etc. and is not relevant to the choice of reflections for refinement. R-factors based on F^2^ are statistically about twice as large as those based on F, and R- factors based on ALL data will be even larger.

### Fractional atomic coordinates and isotropic or equivalent isotropic displacement parameters (Å^2^)

**Table:**

	*x*	*y*	*z*	*U*_iso_*/*U*_eq_	
Zn1	0.057453 (6)	0.825871 (11)	0.151068 (9)	0.03470 (4)	
Cl1	0.094347 (18)	0.82859 (2)	0.04109 (2)	0.04377 (6)	
Cl2	0.121812 (17)	0.94430 (3)	0.29852 (2)	0.04885 (7)	
N1	0.03795 (5)	0.63755 (8)	0.17124 (6)	0.03277 (14)	
N2	−0.01309 (5)	0.43948 (8)	0.11361 (7)	0.03566 (16)	
H1N2	−0.0400	0.3776	0.0772	0.043*	
N3	−0.05430 (5)	0.86317 (8)	0.06474 (7)	0.03630 (16)	
H1N3	−0.0613	0.9453	0.0395	0.044*	
H2N3	−0.0606	0.8661	0.1203	0.044*	
C1	0.08602 (5)	0.54368 (9)	0.24599 (8)	0.03401 (16)	
C2	0.15487 (6)	0.55968 (12)	0.34379 (9)	0.0441 (2)	
H2A	0.1765	0.6429	0.3686	0.053*	
C3	0.18917 (8)	0.44533 (15)	0.40179 (11)	0.0548 (3)	
H3A	0.2351	0.4519	0.4673	0.066*	
C4	0.15673 (9)	0.32000 (14)	0.36457 (13)	0.0578 (3)	
H4A	0.1816	0.2457	0.4063	0.069*	
C5	0.08893 (8)	0.30296 (12)	0.26782 (11)	0.0487 (3)	
H5A	0.0677	0.2195	0.2428	0.058*	
C6	0.05428 (6)	0.41848 (9)	0.20999 (8)	0.03638 (18)	
C7	−0.02133 (5)	0.57180 (8)	0.09356 (7)	0.03092 (15)	
C8	−0.08750 (5)	0.62967 (9)	−0.00210 (7)	0.03133 (15)	
C9	−0.13949 (6)	0.54428 (11)	−0.08355 (9)	0.0409 (2)	
H9A	−0.1313	0.4531	−0.0751	0.049*	
C10	−0.20257 (7)	0.59227 (14)	−0.17600 (10)	0.0501 (3)	
H10A	−0.2366	0.5336	−0.2286	0.060*	
C11	−0.21502 (7)	0.72704 (15)	−0.19038 (10)	0.0556 (3)	
H11A	−0.2567	0.7598	−0.2535	0.067*	
C12	−0.16525 (7)	0.81339 (12)	−0.11062 (10)	0.0478 (3)	
H12A	−0.1741	0.9043	−0.1203	0.057*	
C13	−0.10222 (5)	0.76671 (9)	−0.01632 (7)	0.03314 (16)	

### Atomic displacement parameters (Å^2^)

**Table:**

	*U*^11^	*U*^22^	*U*^33^	*U*^12^	*U*^13^	*U*^23^
Zn1	0.03589 (6)	0.02529 (5)	0.03528 (6)	−0.00291 (3)	0.01612 (5)	−0.00044 (3)
Cl1	0.05837 (16)	0.03021 (11)	0.04711 (13)	−0.00299 (9)	0.03297 (13)	0.00024 (8)
Cl2	0.05149 (15)	0.04321 (13)	0.04471 (13)	−0.01328 (11)	0.02364 (11)	−0.01315 (10)
N1	0.0341 (3)	0.0252 (3)	0.0324 (3)	−0.0009 (3)	0.0154 (3)	0.0028 (2)
N2	0.0392 (4)	0.0235 (3)	0.0404 (4)	−0.0010 (3)	0.0207 (3)	0.0010 (3)
N3	0.0372 (4)	0.0252 (3)	0.0366 (4)	0.0020 (3)	0.0156 (3)	−0.0006 (3)
C1	0.0358 (4)	0.0293 (4)	0.0345 (4)	0.0030 (3)	0.0188 (3)	0.0054 (3)
C2	0.0390 (5)	0.0421 (5)	0.0387 (5)	0.0026 (4)	0.0153 (4)	0.0045 (4)
C3	0.0460 (6)	0.0552 (7)	0.0454 (6)	0.0125 (5)	0.0162 (5)	0.0139 (5)
C4	0.0568 (7)	0.0461 (7)	0.0588 (7)	0.0189 (5)	0.0266 (6)	0.0219 (5)
C5	0.0541 (6)	0.0299 (4)	0.0586 (7)	0.0101 (4)	0.0306 (6)	0.0126 (4)
C6	0.0405 (4)	0.0275 (4)	0.0413 (4)	0.0039 (3)	0.0237 (4)	0.0055 (3)
C7	0.0345 (4)	0.0238 (3)	0.0331 (4)	−0.0006 (3)	0.0187 (3)	0.0011 (3)
C8	0.0316 (4)	0.0280 (3)	0.0313 (3)	−0.0009 (3)	0.0163 (3)	−0.0002 (3)
C9	0.0391 (5)	0.0345 (4)	0.0406 (5)	−0.0050 (4)	0.0180 (4)	−0.0065 (4)
C10	0.0374 (5)	0.0505 (6)	0.0421 (5)	−0.0056 (4)	0.0112 (4)	−0.0100 (5)
C11	0.0369 (5)	0.0533 (7)	0.0440 (6)	0.0034 (5)	0.0045 (4)	−0.0008 (5)
C12	0.0372 (5)	0.0385 (5)	0.0438 (5)	0.0061 (4)	0.0095 (4)	0.0038 (4)
C13	0.0307 (4)	0.0288 (4)	0.0334 (4)	0.0015 (3)	0.0147 (3)	0.0007 (3)

### Geometric parameters (Å, °)

**Table:**

Zn1—N1	2.0068 (8)	C3—H3A	0.9300
Zn1—N3	2.0471 (9)	C4—C5	1.381 (2)
Zn1—Cl2	2.2041 (3)	C4—H4A	0.9300
Zn1—Cl1	2.2589 (3)	C5—C6	1.3933 (14)
N1—C7	1.3300 (12)	C5—H5A	0.9300
N1—C1	1.3890 (12)	C7—C8	1.4668 (12)
N2—C7	1.3554 (12)	C8—C9	1.4009 (13)
N2—C6	1.3798 (13)	C8—C13	1.4049 (13)
N2—H1N2	0.8188	C9—C10	1.3797 (16)
N3—C13	1.4460 (12)	C9—H9A	0.9300
N3—H1N3	0.8877	C10—C11	1.376 (2)
N3—H2N3	0.9418	C10—H10A	0.9300
C1—C6	1.3916 (14)	C11—C12	1.3816 (18)
C1—C2	1.3978 (15)	C11—H11A	0.9300
C2—C3	1.3830 (17)	C12—C13	1.3878 (14)
C2—H2A	0.9300	C12—H12A	0.9300
C3—C4	1.399 (2)		
			
N1—Zn1—N3	89.36 (3)	C3—C4—H4A	118.9
N1—Zn1—Cl2	114.83 (3)	C4—C5—C6	115.92 (12)
N3—Zn1—Cl2	112.74 (3)	C4—C5—H5A	122.0
N1—Zn1—Cl1	109.18 (3)	C6—C5—H5A	122.0
N3—Zn1—Cl1	109.27 (3)	N2—C6—C1	105.68 (8)
Cl2—Zn1—Cl1	117.844 (13)	N2—C6—C5	131.80 (10)
C7—N1—C1	106.79 (8)	C1—C6—C5	122.52 (10)
C7—N1—Zn1	121.20 (6)	N1—C7—N2	110.58 (8)
C1—N1—Zn1	129.89 (7)	N1—C7—C8	126.51 (8)
C7—N2—C6	108.33 (8)	N2—C7—C8	122.91 (8)
C7—N2—H1N2	130.1	C9—C8—C13	117.84 (9)
C6—N2—H1N2	121.5	C9—C8—C7	118.62 (9)
C13—N3—Zn1	116.24 (6)	C13—C8—C7	123.53 (8)
C13—N3—H1N3	113.1	C10—C9—C8	121.57 (10)
Zn1—N3—H1N3	107.2	C10—C9—H9A	119.2
C13—N3—H2N3	112.7	C8—C9—H9A	119.2
Zn1—N3—H2N3	99.8	C11—C10—C9	119.95 (10)
H1N3—N3—H2N3	106.7	C11—C10—H10A	120.0
N1—C1—C6	108.60 (8)	C9—C10—H10A	120.0
N1—C1—C2	130.32 (9)	C10—C11—C12	119.67 (11)
C6—C1—C2	121.07 (9)	C10—C11—H11A	120.2
C3—C2—C1	116.57 (11)	C12—C11—H11A	120.2
C3—C2—H2A	121.7	C11—C12—C13	121.13 (11)
C1—C2—H2A	121.7	C11—C12—H12A	119.4
C2—C3—C4	121.78 (12)	C13—C12—H12A	119.4
C2—C3—H3A	119.1	C12—C13—C8	119.79 (9)
C4—C3—H3A	119.1	C12—C13—N3	117.73 (9)
C5—C4—C3	122.13 (11)	C8—C13—N3	122.48 (8)
C5—C4—H4A	118.9		
			
N3—Zn1—N1—C7	37.88 (8)	C4—C5—C6—C1	0.90 (19)
Cl2—Zn1—N1—C7	152.76 (7)	C1—N1—C7—N2	−1.27 (11)
Cl1—Zn1—N1—C7	−72.34 (8)	Zn1—N1—C7—N2	163.70 (7)
N3—Zn1—N1—C1	−160.99 (9)	C1—N1—C7—C8	178.98 (9)
Cl2—Zn1—N1—C1	−46.11 (10)	Zn1—N1—C7—C8	−16.04 (14)
Cl1—Zn1—N1—C1	88.79 (9)	C6—N2—C7—N1	1.11 (12)
N1—Zn1—N3—C13	−47.06 (7)	C6—N2—C7—C8	−179.14 (9)
Cl2—Zn1—N3—C13	−163.85 (6)	N1—C7—C8—C9	170.17 (10)
Cl1—Zn1—N3—C13	63.07 (7)	N2—C7—C8—C9	−9.55 (15)
C7—N1—C1—C6	0.96 (11)	N1—C7—C8—C13	−10.56 (16)
Zn1—N1—C1—C6	−162.24 (7)	N2—C7—C8—C13	169.73 (10)
C7—N1—C1—C2	−177.97 (12)	C13—C8—C9—C10	1.27 (17)
Zn1—N1—C1—C2	18.82 (17)	C7—C8—C9—C10	−179.41 (11)
N1—C1—C2—C3	178.91 (12)	C8—C9—C10—C11	0.9 (2)
C6—C1—C2—C3	0.09 (18)	C9—C10—C11—C12	−1.9 (2)
C1—C2—C3—C4	0.0 (2)	C10—C11—C12—C13	0.7 (2)
C2—C3—C4—C5	0.3 (3)	C11—C12—C13—C8	1.5 (2)
C3—C4—C5—C6	−0.8 (2)	C11—C12—C13—N3	−177.82 (13)
C7—N2—C6—C1	−0.46 (11)	C9—C8—C13—C12	−2.43 (16)
C7—N2—C6—C5	178.79 (13)	C7—C8—C13—C12	178.29 (11)
N1—C1—C6—N2	−0.30 (11)	C9—C8—C13—N3	176.84 (10)
C2—C1—C6—N2	178.75 (10)	C7—C8—C13—N3	−2.44 (15)
N1—C1—C6—C5	−179.64 (11)	Zn1—N3—C13—C12	−143.37 (10)
C2—C1—C6—C5	−0.59 (18)	Zn1—N3—C13—C8	37.35 (12)
C4—C5—C6—N2	−178.24 (13)		

### Hydrogen-bond geometry (Å, °)

**Table:**

*D*—H···*A*	*D*—H	H···*A*	*D*···*A*	*D*—H···*A*
N2—H1N2···Cl1^i^	0.82	2.56	3.3503 (9)	164
N3—H1N3···Cl1^ii^	0.89	2.49	3.3753 (9)	174
N3—H2N3···Cl2^iii^	0.94	2.44	3.3015 (12)	153

Symmetry codes: (i) −*x*, −*y*+1, −*z*; (ii) −*x*, −*y*+2, −*z*; (iii) −*x*, *y*, −*z*+1/2.
